# Immunotherapy in Squamous Cell Cancer of the Esophagus

**DOI:** 10.3390/curroncol29040200

**Published:** 2022-03-30

**Authors:** Peter Thuss-Patience, Alexander Stein

**Affiliations:** 1 Department of Hematology, Oncology and Cancer Immunology, Charité—University Medicine Berlin, 13353 Berlin, Germany; 2 Hematology-Oncology Practice Eppendorf (HOPE), 20249 Hamburg, Germany; stein@hope-hamburg.de; 3 University Cancer Center Hamburg, University Medical Center Hamburg-Eppendorf, 20251 Hamburg, Germany

**Keywords:** immunotherapy, esophagus, squamous cell carcinoma, ESCC, checkpoint inhibitor

## Abstract

Treatment of esophageal carcinoma has changed dramatically following several landmark trials, which have proven the benefit of immunotherapy. The selective PD-1 (programmed cell death ligand-1)-inhibitor nivolumab has been shown to improve DFS in the adjuvant therapy setting (CheckMate-577). In the first-line treatment, PD-L1 positive (CPS ≥ 10) squamous cell carcinoma patients (pts) have been shown to have an increased OS following treatment with the PD-1-inhibitor pembrolizumab in combination with chemotherapy (KEYNOTE-590). Nivolumab also improved overall survival in the first line setting either combined with ipilimumab or with chemotherapy (CheckMate 648) compared to chemotherapy alone. In Asian first-line patients, phase III trials investigating camrelizumab (ESCORT 1), toripalimab (JUPITER 06), or sintilimab (ORIENT 15) in addition to chemotherapy also showed significant survival benefits. In the second-line setting, monotherapy with nivolumab (ATTRACTION-03), pembrolizumab (KEYNOTE-181), camrelizumab (ESCORT), and tislelizumab (RATIONALE 302) demonstrated a benefit in OS in comparison to chemotherapy. Here we will review these trials and integrate them into the current treatment algorithm.

## 1. Introduction

Esophageal cancer causes approximately 604,000 new cases globally and belongs to the 10 most common new cancer cases worldwide. About 544,000 deaths were estimated globally in 2020 [1]. With an incidence of 3.1%, the mortality is higher than in other cancers (5.5%) [1]. Esophageal cancer is most common in Eastern Asia with an incidence of 18.2 in males and 6.8 in females, whereas in Western Europe the incidences are 6.6 and 1.8, respectively [1]. Worldwide esophageal squamous cell carcinoma (ESCC, mainly proximal esophagus) accounts for 90% of all esophageal carcinomas and esophageal adenocarcinoma (EAC) for 10% [2]. In this review, we concentrate on esophageal squamous cell cancer.

Before the era of checkpoint-inhibition in esophageal cancer, the treatment algorithms were based on neoadjuvant radiochemotherapy followed by surgery, definitive chemoradiation, and systemic chemotherapy for metastatic disease. According to the CROSS trial [3,4], patients with locally advanced resectable esophageal cancer are treated with a preoperative radiochemotherapy consisting of five weekly cycles of carboplatin and paclitaxel with concurrent radiotherapy (41.4 Gy in 23 fractions) followed by surgery. The absolute 10-year overall survival benefit was 13% (38% vs. 25%). Neoadjuvant chemoradiotherapy reduced the risk of death from esophageal cancer (HR, 0.60; 95% CI, 0.46 to 0.80). The benefit was highest for squamous cell carcinoma with 10-year overall survival rates of 46%, versus 23% with surgery only. In the subgroup of adenocarcinoma, 10-year OS rates were 36% versus 26%. The benefit arose from the reduction of locoregional recurrences, whereas the rate of distant metastases was not reduced. [3,4]. In the palliative setting, systemic chemotherapy is based on platin and 5-FU [5], whereas in Asia, platin and paclitaxel are frequently used. [6] With systemic treatment in the advanced metastatic setting, median OS ranges about 10 months in the west [5] and 12 months in Asia [6].

After failure of a palliative first-line treatment, there are no randomized trials that prove the benefit of chemotherapy compared with best supportive care (BSC) for squamous cell carcinoma. In patients with a good performance status irinotecan, paclitaxel or docetaxel are frequently used.

Immune checkpoint inhibitors have changed the treatment algorithm of esophageal squamous cell cancer dramatically. The programmed death receptor 1 (PD-1) and its ligand (PD-L1) play a crucial role in modulating the immune response against cancer cells. When cytotoxic T-cells bind via their PD-1 receptor to its ligand PD-L1 on cancer cells, these cells have the ability to evade the anti-tumor immune response. Expression of the CTLA-4 receptor on T-cells may further promote immune escape. Cancer cell mediated upregulation of CTLA-4 on T-cells augments the recruitment of immunosuppressive T-cells.

Antibodies against the checkpoint proteins PD-1 /PD-L1 and CTLA-4 may interrupt this process of immune escape and reactivate anti-cancer T-cell activity [7]. The level of expression of PD-L1 is a predictive biomarker for the effectivity of the antibodies against PD-L1. PD-L1 levels of expression on cancer cells can be determined immunohistochemically and is reported as the tumor proportion score TPS, on immune cells, reported as IC, and calculated for both sides as the combined positivity score (CPS).

These biomarkers may identify those patient subgroups benefiting from single agent PD-1/PD-L1 inhibitors, whereas most patients require combination strategies. Beside the current treatment landscape in squamous cell cancer of the esophagus combining PD-1/PD-L1 inhibitors with chemotherapy, combinations with targeted agents have shown promising data in other tumor types or have even been approved (e.g., in renal cell or endometrial cancer). Current combination strategies are applied biomarker based (e.g., HER2 positive gastroesophageal adenocarcinoma) or in generally immune-checkpoint inhibitor sensitive diseases without specific biomarkers (e.g., hepatocellular carcinoma). Ongoing trials in squamous cell cancer of the esophagus evaluate combination of tyrosine kinase inhibitors with PD-1/PD-L1 inhibitors (e.g., lenvatinib and pembrolizumab in LEAP 014 trial).

Methodology: In this review we describe and discuss all phase III trials treating esophageal squamous cell cancer with immune checkpoint inhibitors. We include trials published or presented at a major conference.

Table 1 shows a summary of the phase III trials conducted on esophageal cancer.

## 2. Curative Setting

In the perioperative setting adjuvant treatment with nivolumab is integrated in the current treatment algorithm based on the CheckMate 577 study [8]. In this study, 794 patients with esophageal or gastroesophageal junction carcinoma, following neoadjuvant chemoradiation therapy and RO-resection with residual disease in terms of non-pathological complete remission (≥ypT1 and/or ≥ypN1), were randomized between adjuvant nivolumab versus placebo. In total, 532 patients received nivolumab and 262 placebo. Of them, 71% of patients had adenocarcinoma (AC) and 29% squamous cell carcinoma; 14% of patients in the nivolumab group came from Asia, 60% of tumors were located in the esophagus, and 40 % in the gastroesophageal junction. The median disease-free survival (DFS) was 22.4 months (95% confidence interval [CI], 16.6 to 34) among patients who received nivolumab and 11 months (95% CI, 8.3 to 14.3) among those who received placebo (hazard ratio for disease recurrence or death, 0.69; 96.4% CI, 0.56 to 0.86; *p* < 0.001). DFS was significantly improved in both histology subtypes with a median of 19.4 versus 11.1 mths hazard ratio (HR) 0.75 (95% CI 0.59–16.8) in adenocarcinoma and 19.7 versus 11.0 mths (HR 0.61 (95% CI 0.42–0.88) in ESCC. In total, 16% of patients had a tumor-cell PD-L1 expression of ≥1%. The tumor-cell PD-L1 expression level (TPS) was not predictive for benefit in this trial. In contrast, the combined positivity score (CPS) (cut off 5) better predicted the benefit of adjuvant nivolumab with a HR of 0.6 in CPS ≥ 5 (56% of patients) compared to HR 0.85 in CPS < 5 [17].

**Figure 1:**
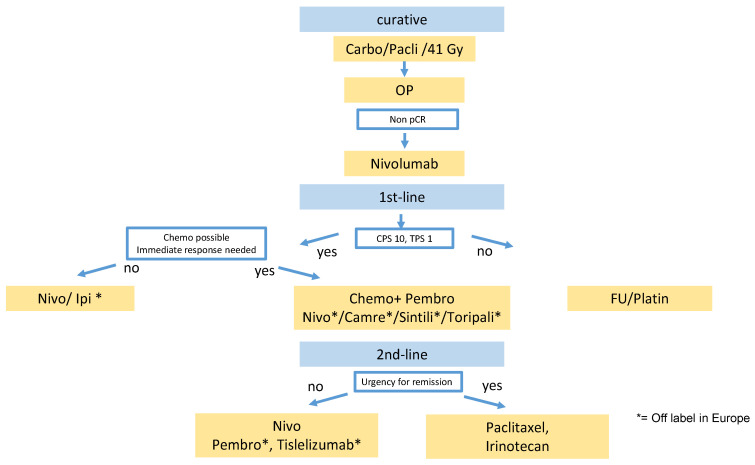
Proposed Treatment Algorithm for Esophageal Squamous Cell Carcinoma Carbo = carboplatin, Pacli = paclitaxel, Nivo = nivolumab, Ipi = ipilimumab, Pembro = pembrolizumab, FU = fluoropyrimidine, Camre = camrelizumab, Sintili= sintilimab, Toripali = toripalimab.

Notably, only patients with a poor prognosis in terms of residual viable tumor cells after neoadjuvant radiochemotherapy were included in this trial. Complete response after neaodjuvant radiochemotherapy was noted in 49% of ESCC and 23% of EAC in the CROSS trial [4]. Based on the trial design, it remains unclear whether these patients would also benefit from adjuvant nivolumab. In Europe, adenocarcinoma of the gastroesophageal junction (GEJ) are frequently treated with perioperative FLOT chemotherapy based on the AIO FLOT-4 trial [18]. It is unclear whether perioperative FLOT is as effective as preoperative radiochemotherapy and adjuvant nivolumab for GEJ adenocarcinoma. Furthermore, several ongoing trials will evaluate the addition of PD-1 inhibitors to perioperative FLOT and will likely further raise the efficacy of perioperative systemic treatment (AIO DANTE, KEYNOTE 585, MATTERHORN). So far, there are no OS data for adjuvant nivolumab. In contrast to neoadjuvant radiochemotherapy alone as in the CROSS trial (see above), nivolumab also prolongs distant metastases-free survival. Based on these data, nivolumab was approved for adjuvant treatment of ESCC and EAC of the esophagus after neoadjuvant radiochemotherapy and R0 resection with incomplete response without limitations by PD-L1 positivity, despite the rather low efficacy in the CPS < 5 subgroup (Figure 1).

In the setting of locally advanced/inoperable disease, ongoing trials evaluate the addition of PD-1 inhibitors to chemoradiation both in neoadjuvant and definitive settings.

## 3. Palliative First-Line Setting

In ESCC and EAC, pembrolizumab plus chemotherapy achieved an improvement of overall survival compared with chemotherapy alone [9]. The KEYNOTE-590 trial was a randomized double-blind international study that compared pembrolizumab plus chemotherapy (Cisplatin + 5-FU) with chemotherapy alone. In total, 749 patients (73% ESCC and 25% EAC) with either locally advanced or with metastatic oesophageal carcinoma (including Siewert type 1 adenocarcinoma of the esophago-gastric junction) were randomized 1:1.

Of them, 53% of patients were Asian. In the ITT population of all patients regardless of CPS and tumor histology, there is a benefit in OS in the combination group of pembrolizumab plus chemotherapy (OS all patients 12.4 vs. 9.8 months, HR 0.73 (95% CI 0.62–0.86, *p* < 0.0001); PFS all patients 6.3 vs. 5.8 months, HR 0.65 (95% CI 0.55–0.76). Notably, the benefit of adding pembrolizumab to chemotherapy is confined to EAC and ESCC with a PD-L1 CPS ≥ 10 (51% of patients) with a HR 0.62 (95% CI 0.49–0.78); *p* < 0.0001), whereas patients with a CPS < 10 (47% of patients) do not benefit with statistical significance (HR 0.86 (95% CI 0.68–1.10)). In addition, treatment benefit seems to be pronounced in the ESCC CPS ≥ 10 cohort (HR 0.57 (95% CI, 0.43–0.75)) compared to CPS < 10 (HR 0.99 (95% CI, 0.74–1.32)). For EAC, the cohorts are relatively small (about 50 patients) and thus the data are far less clear, likely because of methodological rather than medical reasons, with HR 0.83 in the CPS ≥ 10 cohort (95% CI, 0.52–1.34) compared to HR of 0.66 in the CPS < 10 cohort (95% CI, 0.74–1.32).

Based on these data, pembrolizumab is approved by the EMA for ESCC and adenocarcinoma of the GEJ in combination with a platin- and fluoropyrimidine-based chemotherapy with a CPS ≥ 10.

Thus, pembrolizumab is as a standard treatment option for ESCC and EAC with a CPS 10 or above in the current treatment algorithm.

Nivolumab has also been shown to prolong OS in ESCC, in addition to cisplatin/-5-FU or Ipilimumab versus chemotherapy alone [10]. In the Checkmate 648 randomized phase III trial, presented at ASCO 2021, 970 patients with unresectable advanced or metastatic ESCC were randomized between nivolumab and cisplatin/5-FU versus nivolumab 3 mg/kg + ipilimumab 1 mg/kg versus cisplatin/5-FU. In total, 70 % of patients were Asian, 30% non-Asian, tumor cell PD-L1 expression was ≥1% in 49% of patients and <1% in 51% of patients. Comparing chemotherapy plus nivolumab with chemotherapy alone for the primary endpoint (tumor cell PD-L1 (TPS) ≥ 1%, *n* = 315) OS was significantly prolonged (median OS 15.4 versus 9.1 mths, HR 0.54; 99.5% CI 0.37–0.80; *p* < 0.0001). OS rate after 1 year favored the nivolumab arm (37% versus 58%). Of note, the OS benefit was limited to patients with a TPS ≥ 1. For patients with a TPS < 1% the HR was 0.98. Comparing nivolumab + ipilimumab with chemotherapy alone for the primary endpoint (TPS ≥ 1%; *n* = 315), there was also a significant OS benefit for patients receiving immunotherapy (med OS 13.7 mths versus 9.1 mths; HR 0.64; 98.6% CI 0.46–0.90) with 57 versus 37% of patients being alive at 12 months. Similar to the chemotherapy combination, there was no benefit for the 330 patients with a TPS < 1% (HR: 0.96). Notably, the survival curves cross at about 6 months, a pattern often seen in immunotherapy regimen compared to chemotherapy. Further analyses are needed to reliably identify, before treatment initiation, those patients who will not benefit from immunotherapy. The subgroup of females also did not seem to benefit from the nivolumab/ipilimumab combination with a HR of 1.36 favoring chemotherapy, but multivariate analyses are needed to determine whether, in fact, gender or indeed other factors (e.g., smoking, TPS expression level, CPS, better response to chemotherapy in females, etc.) can explain this non-benefit under therapy with this combination.

Approval for nivolumab for patients with ESCC and TPS ≥ 1% is expected. Further CPS-based subgroup analyses may be helpful choosing among the available regimens.

Camrelizumab, a PD-1 antibody investigated mainly in China, may prove OS benefit in addition to paclitaxel 175 mg/m² /cisplatin 75 mg/m² [6]. In the ESCORT 1 trial, 595 Chinese patients with ESCC were included, and 55% of patients had a TPS ≥ 1% in their tumors. The OS survival for all patients, including all TPS, was significantly prolonged (HR 0.70, 95% CI 0.56–0.88, *p* = 0.001). Subgroup analyses show that those patients with a PD-L1 TPS ≥ 1% had a HR of 0.59 (95% CI 0.43–0.80) compared to 0.79 (95% CI 0.57–1.11) for those with a TPS < 1%. TPS is predictive for the degree of benefit. Similarly, a CPS analysis would be interesting.

Sintilimab, another PD-1 antibody, has also been investigated in Asia [11]. In total, 659 Asian patients (98% Chinese) were randomized between sintilimab and chemotherapy versus chemotherapy alone, presented at ESMO 2021. Chemotherapy was either paclitaxel (175 mg/m²)/cisplatin (75 mg/m²) or cisplatin (75 mg/m²) and 5-FU 800 mg/m² d1–5. Of them, 58% of patients had a CPS ≥ 10. OS was significantly prolonged in patients CPS ≥ 10 (med OD 13.6 versus 17.2 mths; HR 0.638, 95% CI 0.480–0.848, *p* = 0.0018) and in all patients (med OS 12.5 versus 16.7 mths, HR 0.628, 95% CI 0.508–0.777), *p* < 0.0001). In this trial, in which data for TPS and CPS are presented, the benefit is not dependent on the TPS nor the CPS expression level. The reason for this is unclear because this is the only trial not showing any predictive value of PD-L1 expression.

Toripalimab, a humanized monoclonal PD-1 antibody investigated in China, may also show survival benefit in a randomized phase III (JUPITER 06) as first-line treatment in 659 Chinese ESCC patients, presented at ESMO 2021 [12]. Toripalimab was added to Paclitaxel 175 mg/m²/cisplatin 75 mg/m² and compared with chemotherapy only. Med OS was improved from 11 to 17 mths (HR 0.58, 95% CI 0.425–0.783, *p* = 0.00036). Of them, 78% of patients had a CPS ≥ 1; 45% of patients in the toripalimab group and 38% of patients in the placebo group had a CPS ≥ 10. Data whether CPS 10 predicts for benefit have not yet been presented.

These phase III trials of camrelizumab, sintilimab, and toripalimab did not include Caucasian patients. As, so far, there is no evidence that Asian patients respond differently to checkpoint inhibitors compared to Caucasians, these trials also support the beneficial class effect of checkpoint inhibitors for ESCC.

## 4. Palliative Second-Line Setting

Pembrolizumab has been investigated in the second-line setting. In the KEYNOTE 181 trial [13], 628 patients were randomized between monotherapy with pembrolizumab and chemotherapy according to investigators’ choice (paclitaxel, docetaxel, or irinotecan). Of the patients included, 38% were of Asian origin, 63% had ESCC, and 36% EAC, and 35% had an PD-L1 CPS score ≥ 10. Pembrolizumab versus chemotherapy was shown to improve OS in patients with CPS ≥ 10 (median, 9.3 vs. 6.7 mths; HR: 0.69; 95% CI, 0.52 to 0.93; *p* = 0.0074). Median OS was 8.2 mths versus 7.1 mths (HR, 0.78; 95% CI, 0.63 to 0.96]; *p* = 0.0095) in patients with squamous cell carcinoma and 7.1 mths versus 7.1 mths (HR, 0.89; 95% CI, 0.75 to 1.05; *p* = 0.0560) in all patients. The estimated 12-month OS rate was 43% (95% CI, 33.5% to 52.1%) with pembrolizumab versus 20% (95% CI, 13.5% to 28.3%) with chemotherapy.

Looking at subgroup analyses, CPS ≥ 10 was predictive for benefit with a HR of 0.7 (95% CI 0.52–0.93) versus HR 1 (59% CI 0.81–1.24) for CPS < 10. Adenocarcinomas, even with a CPS ≥ 10, did not seem to benefit, although it was a group of only 56 patients (HR 0.93, 95% CI 0.52–1.65). The benefit was also less in patients from outside Asia, regardless of whether the CPS was equal to or above 10, or whether they had ESCC histology. Due to these reasons, pembrolizumab was approved by the FDA for ESCC with a CPS ≥ 10, but not approved by the EMA.

Nivolumab has been investigated in the second-line setting in the ATTRACTION 03 trial [14]. In total, 419 patients with advanced ESCC refractory or intolerance to previous chemotherapy were randomized in this multicenter phase III trial. Treatment consisted either of nivolumab monotherapy or chemotherapy (paclitaxel or docetaxel). In total, 96% of patients were of Asian origin. Independent of PD-L1-expression rate, a significant benefit in OS of nivolumab compared with chemotherapy (median OS 10.9 vs. 8.4 months, HR 95% CI: 0.77 (0.62–0.96), *p* = 0.019) was shown. At 12 months, 47% of patients were alive in the nivolumab arm, compared with 34% in the chemotherapy arm. In the PD-L1 unselected population, there was a crossing in the OS Kaplan-Meier curves in the first 5 months with more patients dying if on nivolumab monotherapy. It is currently unclear how to identify these patients prior to treatment. Comparing PD-L1 expression groups, the benefit seems to be more pronounced in the TPS ≥ 1 group (TPS ≥ 1: HR 0.69, 95% CI 0.51–0.94; TPS < 1: HR: 0.84; 95% CI 0.62–1.14). In addition, the crossing of the curves is less in the PD-L1 TPS ≥ 1 population. Nivolumab has been approved in Europe for all ESCC after failure to platin/5-FU, regardless of PD-L1 expression.

In our treatment algorithm (Figure 1), we offer nivolumab preferably to patients after platin/5-FU whose tumor has some PD-L1 expression and favor chemotherapy if there is no PD-L1 expression in TPS or CPS.

Camrelizumab has been investigated in the ESCORT trial in the second-line setting in a Chinese population [15]. In total, 457 patients were randomly assigned to camrelizumab or chemotherapy (docetaxel or irinotecan). With a median follow-up time of 8.3 months (IQR 4.1–12.8) in the camrelizumab group and 6.2 months (3.6–10.1) in the chemotherapy group, median overall survival was 8.3 months (95% CI 6.8–9.7) in the camrelizumab group and 6.2 months (5.7–6.9) in the chemotherapy group (hazard ratio 0.71, 95% CI 0.57–0.87; *p* = 0.0010).

Tislelizumb, a monoclonal antibody against PD-1, has also been investigated in patients after failure of a prior platin/fluoropyrimidine-based chemotherapy. In the RATIONALE 302 trial [16], 512 patients were randomized between tislelizumab monotherapy versus paclitaxel, docetaxel, or irinotecan (investigator’s choice), and 21% of patients came from Europe or the USA. OS was significantly prolonged with a median OS of 8.3 mths versus 6.3 mths (HR: 0.70; 95% CI 0.57–0.85, *p* = 0.0001). In this trial, there was no crossing of the OS curves seen. This benefit was more pronounced in the subgroup of patients with a higher PD-L1 expression (CPS ≥ 10) (HR: 0,53 95% CI 0,37–0,77), compared to CPS < 10 (HR: 0.85, 95% CI 0.65–1.11).

## 5. Discussion

There are not many diseases in which so many positive phase III trials have recently changed the treatment landscape. Checkpoint inhibitors are now an integral part of the treatment algorithm in esophageal squamous cell carcinoma (Figure 1).

In the perioperative setting, nivolumab prolongs DFS after radiochemotherapy and surgery. In the first-line palliative setting, we have five positive phase III trials. Pembrolizumab is approved for CPS ≥ 10 combined with platin/fluropyrimidine. OS prolongation was also proven for nivolumab together with platin/5FU and together with ipilimumab, and for camrelizumab, sintilimab, or toripalimab in an Asian population combined with platin and paclitaxel. Table 2 summarizes the current approval situation in Europe and the U.S.

Checkpoint inhibitors are in general better tolerated than chemotherapy but they still have their own toxicity profile. In the monotherapy trials, the checkpoint specific toxicity can be seen best. The KEYNOTE 181 trial [13] may give a representative impression of the frequency of adverse events. With pembrolizumab, grade 3–5 adverse events were observed in 18% of patients. Hypothyroidism (11.5%) or hyperthyroidism (4.1%) are the most common side effects and can usually be managed easily. However, more severe side effects like pneumonitis (4.8%), hepatitis (1.9%), or colitis (0.9%) may also occur. The more we use checkpoint inhibitors, the more we may also need to watch out for rare side effects like hypophysitis, encephalitis, or autoimmune neuropathy.

In several trials with checkpoint inhibitors without chemotherapy, we observe a crossing of OS curves as a sign of temporary inferiority compared to chemotherapy for some patients (ATTRACTION 03; CheckMate 648 Nivolumab/Ipilimumab arm) [10,14]. It is currently unclear which patients have a dismal effect. Potentially those with fast progressing tumors may rather need upfront chemotherapy to immediately control the disease. Further analyses of the different trials are urgently needed.

There is also debate over whether Asians and Caucasians have different amounts of benefit from checkpoint inhibition. Comparing the HR in the different trials between Asian and Caucasian patients for OS, no consistent pattern can be seen: HR Asian versus Caucasian in CheckMate 577 (0.70 vs. 0.71); KEYNOTE 590 (0.64 vs. 0.63); CheckMate 648 (Chemo/Nivo: 0.74 vs. 0.74, Ipi/Nivo: 0.83 vs. 0.69); KEYNOTE 181: (0.59 vs. 0.83); ATTRACTION 03: (0.73 vs. 0.53 (small numbers)); RATIONALE 302 (0.72 vs. 0.53). [8,9,10,13,14,16]. Therefore, currently there is no indication that Asians have a different benefit from checkpoint inhibition than Caucasians.

In our proposed treatment algorithm (Figure 1), we included checkpoint inhibitors now in the adjuvant, first-line, and second-line settings. Currently, it is unclear whether re-exposure to a checkpoint inhibitor is beneficial after progression during checkpoint-inhibitor treatment. Further trials are needed to evaluate the effect of checkpoint inhibition beyond progression, perhaps in combination with new synergistic immune modulators.

Overall, the integration of immuno-oncology into the treatment of ESCC has revolutionized the treatment algorithms and a big step forward could be achieved for our patients.

Further analyses have to better define those subgroups that benefit the most by developing more precise biomarkers that detect those who benefit even better than PD-L1 expression levels.

## 6. Conclusions

The integration of immune checkpoint inhibition into the treatment algorithm of esophageal squamous cell carcinoma marks a new era of treatment of this dismal disease. Further research on optimizing biomarkers and intelligent immune therapy combinations that augment each other is the agenda for the future.

## Figures and Tables

**Table 1:** Overview of immune checkpoint inhibitor clinical trials in esophageal carcinoma.

Entity	Author	Ref.	Trial	Phase	Treatment	N	Histology	PD-L1 Score	Subgroup	Results Hazard Ratio (OS)
**Curative**										
	Kelly et al., 2021	[8]	Checkmate-577	III	Adjuvant nivo vs. placebo	794	ESCC (30%), EAC (70%)	all comers		DFS HR 0.69 (96.4% CI 0.56–0.86),
								TPS ≥ 1%		DFS HR 0.75 (95% CI 0.45–1.24)
								TPS < 1%		DFS HR 0.73 (95% CI 0.57–0.92)
**First-line**										
	Sun et al., 2021	[9]	Keynote-590	III	Pembro+cis/5-FU vs. cis/5-FU alone	749	ESCC (73%), EAC (27%)	all comers		HR 0.73 (95% CI, 0.62–0.86)
								all comers	ESCC	HR 0.72 (95% CI, 0.60–0.88)
								CPS ≥ 10	ESCC	HR 0.57 (95% CI, 0.43–0.75)
								CPS < 10	ESCC	HR 0.99 (95% CI, 0.74–1.32)
								CPS ≥ 10	EAC	HR 0.83 (95% CI, 0.52–1.34)
								CPS < 10	EAC	HR 0.66 (95% CI, 0.42–1.04)
	Chau et al., 2021	[10]	Checkmate-648	III	Cis/5-FU + nivo vs. ipi+nivo vs. Cis/5-FU	970	ESCC 100%	all comers	Nivo+chemo vs. chemo	HR 0.74 (99.1% CI 0.58–0.96)
								TPS ≥ 1%	Nivo+chemo vs. chemo	HR 0.54 (99.5% CI 0.37–0.80)
								TPS < 1%	Nivo+chemo vs. chemo	HR 0.98
								all comers	Nivo+Ipi vs. chemo	HR 0.78 (98.2% CI 0.62–0.98)
								TPS ≥ 1%	Nivo+Ipi vs. chemo	HR 0.64 (98.6% CI 0.46–0.90)
								TPS < 1%	Nivo+Ipi vs. chemo	HR 0.96
	Luo et al., 2021	[6]	Escort 1	III	Camrelizumab + cis/pacli vs. cis/pacli	595	ESCC 100%	all comers		HR 0.70 (95% CI 0.56–0.88)
								TPS ≥ 1%		HR 0.59 (95% CI 0.43–0.80)
								TPS < 1%		HR 0.79 (95% CI 0.57–1.11)
	Shen et al., 2021	[11]	ORIENT-15	III	Sintilimab + chemo vs. chemo (cis/5-FU or cis/pacli)	659	ESCC 100%	all comers		HR 0.628 (95% CI 0.51–0.78)
								CPS ≥ 10		HR 0.638 (95% CI 0.48–0.85)
								CPS < 10		HR 0.617 (95% CI 0.45–0.85)
	Xu et al., 2021	[12]	JUPITER-06	III	Toripalimab + cis/pacli vs. cis/pacli	514	ESCC 100%	all comers		HR 0.58 (95% CI 0.43–0.78)
								CPS ≥ 1		HR 0.61 (95% CI 0.44–0.87)
								CPS < 1		HR 0.61 (95% CI 0.30–1.25)
**Second-line**										
	Kojima et al., 2020	[13]	Keynote-181	III	Pembro vs. pacli/doce/irino	628	ESCC (64%), EAC (36%)	all comers	ESCC +EAC	HR 0.89 (95% CI, 0.75–1.05)
								all comers	ESCC	HR 0.78 (95% CI, 0.63–0.96)
								CPS ≥ 10	ESCC+EAC	HR 0.69 (95% CI, 0.52–0.93)
								CPS ≥ 10	ESCC	HR 0.64 (95% CI 0.46–0.90)
								CPS < 10	ESCC+EAC	HR 1.00 (95% CI 0.81–1.24)
	Kato et al., 2019	[14]	Attraction-03	III	Nivo vs. Pacli/Doce	419	ESCC 100%	all comers		HR 0.77 (95% CI 0.62–0.96)
								TPS ≥ 1%		HR 0.69 (95% CI 0.51–0.94)
								TPS < 1%		HR 0.84 (95% CI 0.62–1.14)
	Huang et al., 2020	[15]	ESCORT	III	Camrelizumab vs. docetaxel or irinotecan	448	ESCC 100%	all comers		HR 0.71 (95% CI 0.57–0.87)
								TPS ≥ 1%		HR 0.58 (95% CI 0.42–0.81)
								TPS < 1%		HR 0.82 (95% CI 0.62–1.09)
	Shen et al., 2021	[16]	RATIONALE 302	III	Tislelizumab vs. doce/pacli/irino	512	ESCC 100%	all comers		HR 0.70 (95% CI 0.57–0.85)
								CPS ≥ 10		HR 0.53 (95% CI 0.37–0.77)
								CPS < 10		HR 0.85 (95% CI 0.65–1.11)

**Table 2:** Approval of immune checkpoint inhibitor in squamous cell esophageal carcinoma in Europe and the U.S.

Treatmentsetting	Immunotherapeutic Agents	Ref Trial	Approval Europe	Approval US
Adjuvant ypT/N > 0 after chemoradiation and surgery	Nivolumab	CM-577	yes	yes
Firstline	Pembrolizumab (+FU and platinum)	KN-590	yes CPS ≥ 10	yes
	Nivolumab (+FU and platinum)	CM-648	yes TPS ≥ 1	
	Nivolumab + Ipilimumab	CM-648	yes TPS ≥ 1	
Secondline	Pembrolizumab	KN-181		yes CPS ≥ 10
	Nivolumab	Attraction-03	yes	yes
